# Autophagy and Aging: Roles in Skeletal Muscle, Eye, Brain and Hepatic Tissue

**DOI:** 10.3389/fcell.2021.752962

**Published:** 2021-10-28

**Authors:** Ping Li, Yuanzheng Ma, Chengwei Yu, Shoutong Wu, Kai Wang, Hongyang Yi, Weizheng Liang

**Affiliations:** ^1^College of Life Sciences and Health, Institute of Visual Neuroscience and Stem Cell Engineering, Wuhan University of Science and Technology, Wuhan, China; ^2^Department of Physiology, Guangxi University of Chinese Medicine, Nanning, China; ^3^CAS Key Laboratory of Genome Sciences and Information, Beijing Institute of Genomics, Chinese Academy of Sciences, Beijing, China; ^4^School of Future Technology, University of Chinese Academy of Sciences, Beijing, China; ^5^Shenzhen Children’s Hospital, Shenzhen, China; ^6^Harbin Institute of Technology, Harbin, China

**Keywords:** autophagy, aging, age-related diseases, skeletal muscle, eye, brain, liver

## Abstract

Autophagy is an evolutionary conserved degradative process contributing to cytoplasm quality control, metabolic recycling and cell defense. Aging is a universal phenomenon characterized by the progressive accumulation of impaired molecular and reduced turnover of cellular components. Recent evidence suggests a unique role for autophagy in aging and age-related disease. Indeed, autophagic activity declines with age and enhanced autophagy may prevent the progression of many age-related diseases and prolong life span. All tissues experience changes during aging, while the role of autophagy in different tissues varies. This review summarizes the links between autophagy and aging in the whole organism and discusses the physiological and pathological roles of autophagy in the aging process in tissues such as skeletal muscle, eye, brain, and liver.

## Introduction

Autophagy is a tightly orchestrated process that degrades and recycles cytoplasmic components in lysosomes to maintain cellular homeostasis. Although the phenomenon of autophagy was first reported by Thomas P. Ashford and Keith R. Porter in 1962 in the research of lysosomes in rat hepatic cells, the importance of this discovery was not realized (Ashford and Porter, 1962). The concept of autophagy was proposed by Christian de Duve at the international conference of lysosomes in 1963. Driven by Yoshinori Ohsumi, autophagy research had become popular in the 1990s (De Duve and Wattiaux, 1966; Yang and Klionsky, 2010). Reverse-genetic approaches in cell culture and animal models have revealed that quality control, metabolic adaption and cellular defense are three main functions of autophagy (Morishita and Mizushima, 2019; Deretic, 2021). Malfunctioning of autophagy with age may result in systemic diseases such as diabetes, vascular disease and organ-specific pathologies like sarcopenia and neurodegenerative diseases (Schneider and Cuervo, 2014; Levine and Kroemer, 2019). While most previous reviews addressed the nature of autophagy in organism aging, the roles of autophagy in specific tissue aging are less clear. This article seeks to remedy this fragmentation by expanding on the role of autophagy in tissues such as skeletal muscle, eye, brain and liver during aging and its contribution to the according age-related disease.

## Links Between Autophagy and Aging

### The Mechanisms and Functions of Autophagy

**FIGURE 1:**
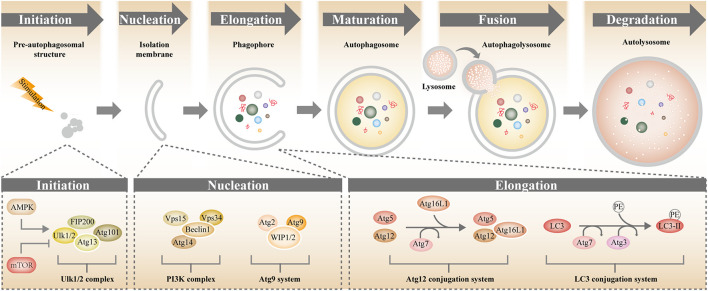
Molecular mechanism of autophagy. Autophagy involves a series of steps including initiation, nucleation, elongation, maturation, fusion, and degradation. AMPK (positive regulator) and mTOR (negative regulator) are the main regulators of autophagy. Functional complexes comprising Atg proteins coordinates and direct the formation of the autophagosome.

Over the last two decades, the molecular mechanisms and physiological functions of autophagy have been extensively studied. Analysis of the delivery route of autophagic cargo to lysosomes has shown that there are at least three types of autophagy: microautophagy, chaperone-mediated autophagy (CMA) and macroautophagy (Yin et al., 2020). In microautophagy, the lysosomal membrane sequesters the cytoplasm in a large and non-specific way and the lysosome degrades the cargo with acidic hydrolases (Schuck, 2020). In CMA, the heat shock cognate protein HSC70 and co-chaperones recognize the proteins bearing conserved KFERQ motif and delivers them to the surface of the lysosome where substrate proteins are translocated into the lysosome lumen by LAMP-2A (lysosomal-associated membrane protein 2A) (Kaushik and Cuervo, 2012). Macroautophagy is the major catabolic mechanism used by eukaryotic cells. In macroautophagy, the cell sequesters the cytosolic components into double-membrane vesicles, the autophagosomes, which subsequently fuse with lysosomes to allow degradation of engulfed substrates by lysosomal hydrolases (Nowak and Edelstein, 2020). Both microautophagy and macroautophagy can be further divided into non-selective autophagy and selective autophagy (Yang et al., 2019). Non-selective autophagy is applied to the turnover of bulk cytoplasm under starvation conditions, whereas selective autophagy is employed in targeting redundant proteins and damaged or aged organelles, including mitochondria, lipid droplets, peroxisomes and so on (Wang et al., 2019). Depending on the cargo being targeted for destruction, selective autophagy can be further categorized into mitophagy (mitochondria), pexophagy (peroxisomes), lipophagy (lipid dropts), ribophagy (ribosomes), aggrephagy (aggregated proteins), and xenophagy (pathogens) (Wang et al., 2019). There are four key steps and over 30 autophagy related genes involved in the process of macroautophagy (Figure 1; Li et al., 2020; Matoba and Noda, 2021). (1) Firstly, the preinitiation complex comprising Atg13, Unc-51 like kinase 1/2 (Ulk1/2) and FAK family-interacting protein of 200 kDa (FIP200) is formed to induce the nucleation of the autophagy-isolation membrane. This process is positively regulated by the upstream energy sensor, Amp-activated protein kinase (AMPK) pathway, and negatively regulated by the nutrient sensor, the mammalian target of rapamycin (mTOR) pathway (Egan et al., 2011; Cicchini et al., 2015). (2) The preinitiation complex then recruits a multi-protein type III phosphoinositide 3 kinases (PI3K) complex, consisting of Atg14, Vps34, and Beclin1, to the rough endoplasmic reticulum (ER) to generate isolation membranes and phagophore (Matsunaga et al., 2010; Cicchini et al., 2015). (3) Subsequently, two ubiquitin-like conjugation systems, the Atg7-Atg3-Atg8/LC3 complex and Atg12-Atg5-Atg16L1 complex, are recruited to the nascent phagophore and induce phagophore elongation and expansion to form the autophagosome (Itakura and Mizushima, 2010; Cicchini et al., 2015). (4) In the final step, the autophagosomes fuse with lysosomes/endosomes to form autolysosomes where digestion happens, which is regulated by small GTPases, lysosomal-associated membrane proteins (Lamp1/2), and the N-ethylmaleimide-sensitive factor attachment protein receptors (SNAREs) (Itakura et al., 2012; Cicchini et al., 2015).

**FIGURE 2:**
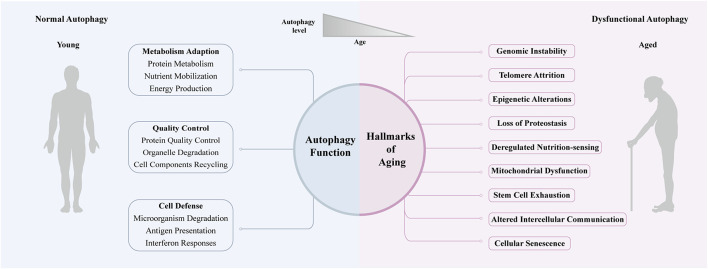
Autophagy and aging. Quality control, metabolic adaption and cellular defense are the three main functions of autophagy, and functional autophagy promotes health during youth. Autophagy declines with aging, which is often associated with the hallmarks of aging and promotes age-related disease.

The best-characterized functions of autophagy are metabolic adaption and quality control wherein protein catabolism was the first well-defined function (Figure 2). The dynamic control of autophagy by nutritional status was the center of early research. Under nutrient deprivation, proteins were mobilized by autophagy to replenish free amino acids and energy (Mortimore and Poso, 1987). Recently, this old function of autophagy has been revisited. Several lines of evidence suggest that in addition to proteolysis, autophagy plays important role in mobilizing various cellular energy stores, such as lipid droplets and glycogen (Kim and Lee, 2014; Morishita and Mizushima, 2019). In nutrient recycling, autophagy is always assumed to be no-selective autophagy. Selective autophagy is employed as a quality-control mechanism to maintain intracellular homeostasis by degrading and recycling cellular components such as aggregative proteins and impaired organelles (Pleet et al., 2018; Morishita and Mizushima, 2019). Autophagic role in maintaining cellular homeostasis soon expanded to host antimicrobial defense. During infection, intracellular microbes are specifically recognized and targeted to autophagosomes for degradation by xenophagy. The intracellular bacteria is often opsonized by ubiquitin or galectin tags and recognized by broad-spectrum selective autophagy receptors such as p62, NBR1, OPTN, and NDP52 (Morishita and Mizushima, 2019). The concept of autophagy as a cell-autonomous defense mechanism was pioneered by a study demonstrating that autophagy could be activated by virus and was targeted by the herpes simplex virus neurovirulence protein (Tallóczy et al., 2002). The systematic recognition of autophagy as a bona fide immunological process was prompted by the discovery that autophagy is capable of eliminating various intracellular bacteria (e.g., *Mycobacterium tuberculosis*, group A *Streptococcus*, *Shigella* and so on) (Gutierrez et al., 2004; Ogawa et al., 2005). Recent studies found that most successful intracellular microbes have evolved intricate mechanisms to circumvent autophagy, which reinforced the antimicrobial significance of autophagy (Deretic, 2021). Since then, autophagy role in immunity has been extended vertically and laterally. Through these functions, autophagy promotes cell fitness, tissue functionality, and longevity.

### Autophagy and the Hallmarks of Aging

Aging is the decline of biological function both at the cellular and organismal level that occurs gradually and continuously. Lopez and colleagues have summarized nine hallmarks of aging: “genomic instability, telomere attrition, epigenetic alterations, loss of proteostasis, deregulated nutrition-sensing, mitochondrial dysfunction, cellular senescence, stem cell exhaustion, and altered intercellular communication” (Lopez-Otin et al., 2013). It’s noteworthy that the malfunctioning of autophagy in old organisms plays a crucial role in these age-related manifestations (Figure 2).

Genomic instability is a prominent feature of aging. It was reported that autophagy can support genomic stability by engulfing and digesting the hazardous cellular components and chromatin fragments and by reducing oxidative stress (Vessoni et al., 2013; Bu et al., 2020). Telomere attrition is another hallmark of aging, which is closely related to low levels of telomerase activity in somatic cells. Notably, induction of autophagy by overexpression of Beclin 1 reduced telomerase activity in Hela cells, which indicated the potential role of autophagy in telomere attrition (Taji et al., 2017). Multiple and progressive epigenetic alterations have emerged as one of the key hallmarks of aging (Folgueras et al., 2018; Gabbianelli and Malavolta, 2018). Studies have shown that autophagy genes can be regulated by various epigenetic modifications and most epigenetic autophagy regulators have been implicated in aging (Aman et al., 2021). Loss of proteostasis resulting from unbalance of protein synthesis, folding and degradation is always linked to aging and aging-related disease. It is widely believed that the anti-aging effect of autophagy is, at least in part, attributed to its capacity to maintain proteostasis by degrading long-lived or damaged proteins (Hansen et al., 2018). Besides, the mTOR pathway (nutrient-sensing pathway) and AMPK pathway (energy-sensing pathway) are well-described regulators of autophagy (Zhang et al., 2014), which emphasized the important role of autophagy in the process of deregulated nutrient-sensing in aging. Mitochondria dysfunction has long been considered as one of the nine hallmarks of aging. The precise role of mitochondria in aging is controversial and complex, but it is increasingly clear that their degradation by mitophagy is crucial for aging and age-related disease. Mitophagy supports mitochondrial quality control not only by the elimination of damaged or superfluous mitochondria but also by the biosynthesis of new ones (Shi et al., 2018). Autophagy is also involved in the regulation of other features of aging, which has been extensively reviewed (Zhang et al., 2016; Barbosa et al., 2018; Stead et al., 2019).

### Autophagy Declines With Age

Recently, several lines of evidence show that aging and autophagy have a bidirectional connection with each other (Figure 2). Autophagy reporter analysis and gene expression studies in many organisms reported that autophagic activity tended to decrease during aging. LC3 is a marker of autophagosomes and autolysosomes. Wilhelm and colleagues examined the autophagic activity in *C. elegans* using fluorescently tagged LGG-1 (ortholog of Atg8/LC3), a marker of autophagosomes and autolysosomes and observed blocked late-stage autophagy in aged worms (Wilhelm et al., 2017). Chang and colleagues conducted a spatiotemporal analysis of autophagy in *C. elegans*. They found that there was an age-associated increase in the number of autophagic vesicles in the intestine, body-wall muscle, pharynx and neurons, which implying the impaired autophagic activity in these tissues (Chang et al., 2017). Moreover, Carnio et al. (2014) monitored the expression level of autophagy markers, such as LC3 and Atg7, in the muscle of mice and humans, and showed that the autophagy system decreased in both species. Numerous studies also demonstrated that the aging rate could be modulated by autophagy. Autophagy seemed to be the nexus of multiple longevity pathways, and environmental or genetic factors affected aging at least partially via regulating autophagy (Rubinsztein et al., 2011). Genetic studies in yeast, worms, flies and mice indicated that related (ATG) genes were required in different extended lifespan models, such as insulin signaling deficiency, caloric restriction and many other longevity paradigms (Hansen et al., 2018). Autophagy induction in these organisms exerted anti-aging effects and improved healthspan (Lopez-Otin et al., 2016; Hansen et al., 2018).

**FIGURE 3:**
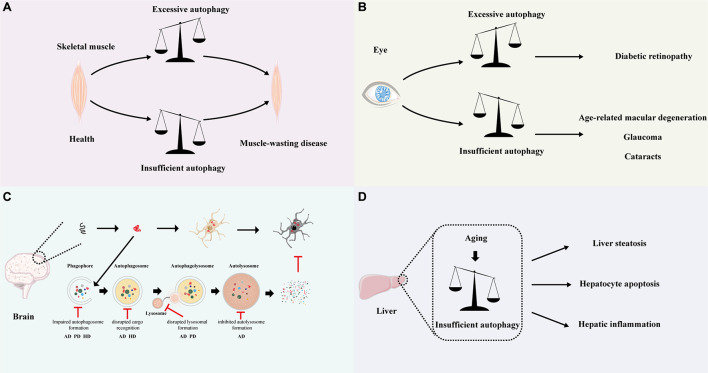
Relationship between autophagy and disease. Schematic depicting the autophagy-related disease in different tissue. **(A)** In the muscle, both excessive and insufficient autophagy will cause muscle-wasting disease. **(B)** In the eye, excessive autophagy may lead to diabetic retinopathy, while insufficient autophagy may result in age-related macular degeneration, glaucoma and cataracts. **(C)** In the brain, the accumulation of misfolded proteins and inclusion bodies is the common pathological hallmark for various neurodegenerative disorders (top). Autophagy may help to eliminate the aggregated proteins and prevent neurodegeneration. Perturbations throughout the autophagic cycle, from autophagosome development to autolysosome formation, have been suggested to cause neurodegenerative disease. The key points in the autophagy pathway along with the associated neurodegenerative diseases are highlighted below. **(D)** Age-related decline in autophagy may lead to liver steatosis, hepatocyte apoptosis, and hepatic inflammation. AD: Alzheimer’s disease; PD: Parkinson’s disease; HD: Huntington disease.

The changes of autophagy during aging and the role of autophagy in regulating lifespan are well-studied, but it remains to be clarified how autophagy in specific tissue impacts tissue-aging and the according age-related disease. Therefore, it is important to understand the tissue-specific role of autophagy in aging, and the autophagy in skeletal muscle, eye, neuron and liver would be reviewed below (Figure 3).

## Autophagy in the Skeletal Muscle and its Role in Aging

Skeletal muscles are important for motion and metabolism, which provide strength for movement and body support and comprise approximately 40% of total-body lean mass (Sakuma et al., 2015). A progressive loss of skeletal muscle mass, strength, and function, a process called sarcopenia, is an inevitable event during aging and contributes to the increased fall incidence and higher mortality in the elderly (Nair, 2005). Therefore, maintaining the cellular homeostasis of skeletal muscle is critical for extending healthspan in humans. It is well-known that skeletal muscle homeostasis is strongly dependent on the balance between the catabolic and anabolic processes. As a vital catabolic process, autophagy is required for skeletal muscle to breaks down the unnecessary old cellular components for rebuilding new cellular architecture. A fine equilibrium of autophagic flux is important for healthy skeletal muscle (Figure 3A). Genetic studies have shown that defective autophagy leads to the degeneration of muscle fiber, which is usually a chronic process and occurs within weeks to months. Atg7 is essential for regulating autophagosome assembly. In mice, knockout Atg7 in muscle led to mitochondrial dysfunction, reticulum distension, disorganized sarcomere, and aberrant concentric membranous structures. Under catabolic conditions, the muscle-specific Atg7-null mice showed neuromuscular junctions (NMJ) instability, higher level of atrophy, muscle loss and degeneration (Masiero et al., 2009). Carnio et al. (2014) demonstrated that aging reduced whereas long-life regular exercise maintained the expression of Atg7 in muscle and overexpression of Atg7 in aged mice improved neuromuscular synaptic function and enhance muscle mass. Epigenetic factors such as histone deacetylases (HDACs) 1 and 2 regulate autophagic flux in skeletal muscle by inducing autophagic gene expression and modulating autophagosomes formation. Moresi and colleagues observed that roughly 40% of mice with muscle-specific deletion of HDAC1 and HDAC2 (dKO mice) died during the perinatal period and exhibited mitochondrial abnormalities and sarcomere degeneration, and dKO mice that survived the first day of life also developed a progressive myopathy, starting from 7 weeks of age (Moresi et al., 2012). Tsc1/2 (Tuberous sclerosis complex 1/2) protein complex negatively regulates mTORC1 (mTORC1, a negative regulator of autophagy). Castets et al. (2013) suggested that skeletal muscle-specific knockout of Tsc1 (TSCmKO) resulted in sustained activation of mTORC1, and blocking the constitutive and starvation-induced autophagy. TSCmKO mice developed a serve, late-onset myopathy and died around 1 year of age; they showed that inhibiting mTORC1 by rapamycin in TSCmKO mice can restore the autophagy flux and ameliorate the myopathy in old TSCmKO mice (Castets et al., 2013). In this scenario, autophagy is protective for skeletal muscle. However, excessive autophagy always causes a rapid decline in muscle mass and muscle atrophy occurs within days to weeks owing to the continued clearance of necessary organelles. Genetic studies showed that muscle-specific inactivation of mTOR led to impaired oxidative metabolism, altered mitochondrial regulation and serve myopathy, resulting in premature death. *Chkb* encodes the choline kinase beta isoform in muscle. Loss-of-function mutations in *Chkb* in mice resulted in mitochondrial dysfunction and mitochondrial loss by elevated mitophagy which lead to rostrocaudal muscular dystrophy (Mitsuhashi et al., 2011). Rev-erb-α is a nuclear receptor that regulating autophagy in muscle by repressing genes involved in autophagosome formation and lysosomal degradation. Woldt et al. (2013) indicated that Rev-erb-α deficiency enhanced autophagy, and leading to increased clearance and impaired mitochondrial function in muscle. The Rev-erb-α deletion mice show severely reduced exercise capacity (Woldt et al., 2013).

Autophagy also plays an anti-aging role in skeletal muscle. Muscle stem cell also referred to as satellite cell usually resides in a quiescent state and is transcriptionally inactive (Schultz, 1978). Autophagy is employed to maintain the stemness of satellite cells and prevent cellular senescence via preserving mitochondrial function. Decreased autophagy in aged satellite cells leads to decreased stem cell fitness, and the re-establishment of autophagy restores their stemness (Garcia-Prat et al., 2016). Constitutive autophagy is also required for active satellite cells (Brack and Rando, 2012). Once satellite cells are activated, they were able to proliferate and replenish the stem cell pool and generate new muscle fibers (Brack and Rando, 2012). Autophagy likely provides energy sources and nutrients for satellite cells activation by degrading unnecessary organelles or proteins. For example, SIRT1, a key nutrient sensor, regulates autophagic flux in satellite cells. Tang and Rando (2014) revealed that deletion of SIRT1 blocks autophagy and led to a delay in satellite cells activation which can be rescued by exogenous pyruvate.

The studies above highlight the importance of autophagy in several aspects of skeletal muscle homeostasis and satellite cells fitness. Next, we will describe the effect of autophagy in skeletal muscle on systemic aging. Increasing evidence suggested that muscle-specific autophagy had been linked to longevity. In *C. elegans*, it has been reported that inhibition of *lgg-1/Atg-8* in the body-wall muscle of adult worms is sufficient to shorten the lifespan of *daf-2* mutants (Chang et al., 2017). In *Drosophila*, muscle-specific overexpression of *Atg8* extends lifespan (Bai et al., 2013). Muscle-derived myokines can modulate systemic aging by targeting different tissues as autocrine, paracrine and endocrine factors. In *Drosophila*, muscle-specific activation of FOXO signaling promoted organism-wide proteostasis during aging, prevented age-related skeletal muscle dysfunction and extended lifespan partially by upregulating basal autophagy (Demontis and Perrimon, 2010). In mice, heterochronic parabiosis or systemic delivery of recombinant growth differentiation factor 11 (GDF11) enhanced basal autophagy and reserved age-related skeletal muscle mass loss and satellite cells dysfunction (Sinha et al., 2014).

## Autophagy in the Eye and its Role in Aging

As a housekeeping process of cellular degradation and recycling, autophagy is crucial for maintaining the physiological function of the eye. Many cells in ocular are highly differentiated non-dividing cells with low cell division rates and high metabolism rates (Rehen et al., 1999). These cells are susceptible to oxidative stress owing to constant exposure to visible light and ultraviolet radiation (Kaarniranta et al., 2013); in response to this oxidative damage, they utilize autophagy for cytoprotection (Frost et al., 2014). In the eye, autophagy-related proteins are widely expressed in various cells, particularly in the retina. Consistent with the expression level, the basal activity of autophagy is high in retina cells, especially in the retinal pigment epithelium (RPE) and photoreceptors, where autophagic responses are induced by light exposure. Moreover, the expression of the autophagy gene is higher during the day than at the night in the retina. For animals kept in constant darkness, the formation of the autophagosomes is greatly reduced in their retina (Reme et al., 1986). Autophagy processes in the phagocytosis of photoreceptor outer segments in RPE cells and are essential for the proper function of these cells (Yao et al., 2014a).

Dysregulated autophagy with age observed in eyes has been proposed to account for the exacerbation of age-related ocular diseases, such as age-related macular degeneration (AMD) and diabetic retinopathy (DR) in the retina, cataracts in the lens, glaucoma in the optic nerve and so on (Pascolini and Mariotti, 2012; Figure 3B). Poor visual function has a significant adverse impact on multiple health aspects, including the activity of daily life, psychological well-being and mortality (Sugita et al., 2020).

Age-related macular degeneration is an irreversible sight-threatening disease featured by the intracellular lipofuscin accumulation of lipofuscin in RPE cells as well as extracellular drusen deposition between RPE and the Bruch’s membrane (BM). In early AMD, the autophagy activity is elevated to compensate for the exacerbate organelles damage caused by increased oxidative stress. Nevertheless, by late AMD, the autophagic system can’t handle the expanded requirements to clear damaged organelles and thus becomes overloaded and dysfunctional (Somasundaran et al., 2020). Compromised autophagy is assumed to contribute to the dysfunction of RPE and the development of AMD. N-retinyl-N-retinylidene ethanolamine (A2E), a prominent toxic lipofuscin component, accumulates in RPE with age. Zhang et al. (2015) revealed that A2E stimulated autophagy in RPE cells in early AMD and elevation of autophagy protected the RPE cells against the adverse effects of A2E by repressing the inflammatory response and decreasing the secretion of VEGFA. Besides, Sayak et al. observed a significant reduction of autophagy proteins in samples from advanced AMD (Mitter et al., 2014).

Diabetic retinopathy (DR), a common diabetic complication, is characterized by the apoptosis of neuron cells and dysfunction of glial cells (Lopes de Faria et al., 2016). DR is one of the leading causes of blindness in adults aged 20–74 years. Growing evidence indicates that the retinal damage in diabetic patients is strongly connected to autophagy. Excessive reactive oxygen species (ROS) provoke pathological autophagy and lead to retinal damage. In normal conditions, ROS stimulates the activation of autophagy to eliminate the damaged mitochondrial to protect the cell (Yao et al., 2014b). Oxidative stress arises when there is an imbalance between the production and elimination of ROS. The increased ROS further damages the mitochondrial DNA and proteins, which in turn induces more ROS, thus creating a vicious cycle (Doblado et al., 2021). Many factors contribute to the increased ROS in DR patients, one of which is hyperglycemia (Catalani et al., 2021). Studies showed that hyperglycemia was the major risk for DR. Hyperglycemia increased mitochondrial reactive oxygen species production, promoted oxidative stress, induced activation of autophagy and eventually resulted in vascular endothelial cell injury (Fernandez-Albarral et al., 2021). In DR, the disruption of the blood-retinal barrier allowed the leakage of cytoplasmic lipoproteins and subsequent lipoprotein modification. The extravasated, modified LDL enhanced ER stress and oxidative stress, activated autophagy and was implicated in the pericyte loss and retinal injury (Fu et al., 2012).

Cataracts are a widespread eye disease in the elders, affecting approximately 20% of adults aged 65 years and older (Schmier et al., 2016). With age, crystallins proteins gradually deposit in the lens and lose their protection function of maintaining lens clarity, which causes lens opacity, light scattering, and ultimately, the development of age-onset cataracts. Autophagy plays a critical role in cataracts. FYCO1 is one of the autophagy genes, which binds LC3, Rab3 and PI(3)P and mediates autolysosome formation (Brennan et al., 2012). Chen et al. (2011) performed genome-wide linkage analysis and found that mutations in FYCO1 were prevalent causes of autosomal recessive congenital cataracts in the Pakistani population. In line with this finding, Kiyotoshi et al. demonstrated that knockout of FYCO1 led to crystallin aggregation and cataracts formation in mice (Satoh et al., 2021). Vps34 (vacuolar protein sorting 34), the catalytic subunit of PI3K (class III phosphatidylinositol 3-kinase) complex, participated in the nucleation of autophagy with an Atg5-independent mechanism. Morishita et al. showed that a lens-specific deletion of Vps34 led to congenital cataracts in mice, while loss of Atg5 in the lens caused age-related cataracts (Costello et al., 2013). In the Atg5-deficiency lens, cortical fiber cells were disorganized and swollen, accompanied by deposition of polyubiquitinated proteins, and insoluble crystallins (Morishita et al., 2013). In conclusion, autophagy is critical in maintaining the transparency of the lens and the disruption of autophagy may contribute to cataract formation.

Glaucoma is a common age-related chronic optic neuropathy with progressive degeneration of retinal ganglion cells (RGCs), resulting in damage to the optic nerve head and a concomitant vision loss (Porter et al., 2013, 2015; Pulliero et al., 2014). Trabecular-meshwork (TM) cells regulate the appropriate intraocular pressure (IOP) by modulating the outflow of aqueous humor, any challenge to TM cells may lead to the development of glaucoma (McMonnies, 2018). In the natural course of aging, oxidative stress accumulates and causes the death of TM cells. To maintain intracellular homeostasis, TM cells utilize autophagy to eliminate the cytotoxic effect of damaged proteins and dysfunctional organelles. However, autophagic activity gradually saturates with age owing to the accumulation of non-degradable substances in the lysosome, which leads to the reduction of lysosome activity. Porter et al. (2013) observed a decreased autophagic activity and an impaired cathepsin B proteolytic maturation in the porcine TM cells subjected to hyperoxia; they speculated that the oxidative stress-induced reduction in autophagic activity may be one of the contributors to the age-related dysfunction of TM cells and may be partly accountable for the pathogenesis of glaucoma. Elevation in IOP is a major risk for glaucoma. In Porter’s another study, they observed an increase in autophagic activity in cultured human TM cells subjected to static biaxial stretch and proved that in porcine TM cells, the high pressure also activated autophagy to cope with the mechanical forces (Porter et al., 2014). In this scene, autophagy is protective for the eye. While Park et al. (2012) demonstrated that in the rat model of chronic hypertensive glaucoma, chronic IOP elevation activated autophagy and led to autophagic cell death of retinal ganglion cells. Normal-tension glaucoma (NTG) is a subgroup of glaucoma, which manifests optic nerve damage without IOP elevation. Several gene mutations are linked to NTG, among them is optineurin (OPTN) (Rezaie et al., 2002). OPTN is an autophagy receptor mediating cargo-selective and non-selective autophagy (Ryan and Tumbarello, 2018). Mutations in the OPTN gene have been linked to the pathogenesis of NTG. E50K and M98K of OPTN are the most common mutations observed in NTG. A study conducted on transgenic mice showed that overexpression of E50K-OPTN activated the Bax pathway and triggered mitophagy, resulting in loss of RGCs with aging (Shim et al., 2017). Sirohi et al. (2013) demonstrated that M98K-OPTN showed a higher coefficient of colocalization with transferrin receptor (TFRC) than wild-type OPTN, hindered the uptake of transferrin and induced RGC-5 death by stimulating the activation of autophagy. The two mutations activated autophagy and led to RGC death with two different mechanisms. Therefore, a better understanding of the roles of autophagy in glaucoma may be helpful to develop novel therapeutic strategies to improve the treatment of this disease.

## Autophagy in the Brain and its Role in Aging

Neurons are postmitotic cells that are unable to dilute the aggregated macromolecular and dysfunctional organelles by cell division and are therefore more susceptible to proteostasis impairment. Autophagy, an alternative cellular degradation pathway, degrades the unnecessary components to achieve cellular homeostasis and is essential for the survival and the proper function of neurons. Numerous pieces of evidence indicate that autophagy declines with age in the brains of several species. Genome-wide analysis reveals that there is a significant transcriptional down-regulation of autophagy during the aging of the human brain (Lipinski et al., 2010). A study on mice also shows aging reduces the protein level of Atg-7, LC3-II and enhances the accumulation of p62 in the total hypothalamic lysates of aged mice and the aged mice phenocopy the metabolic defects observed in POMC neuron-specific *Atg7*-null mice (Kaushik et al., 2012). Age-dependent cognitive decline is inevitable in the aging process, which is observed in both model organisms and humans. Gupta et al. (2013) demonstrate that dietary spermidine protects the *Drosophila* from age-induced memory impairment via autophagy. Proteostasis failure results in protein aggregation and is a defining feature of many age-related neurodegenerative diseases. A growing number of studies suggest that age-related decreases in autophagic activity disrupt neuronal proteostasis and consequently result in the age-dependent onset of neurodegenerative disorders (Figure 3C).

Alzheimer’s disease (AD) is the most prevalent type of dementia in the elderly and is pathologically characterized by the deposition of extracellular amyloid-beta (Aβ) and intracellular hyperphosphorylated tau proteins. TEM studies show that AD is generally accompanied by low or insufficient autophagic activity (Nixon and Yang, 2011; Wolfe et al., 2013). In normal neurons, the majority of newly generated autophagosomes retrograde transport along the axon to the location of the lysosome. However, in AD transgenic mice, it was found that axonal autophagosome vesicle transport was impaired, which may be attributed to the hyperphosphorylated Tau, a microtubule-associated protein (Nilsson et al., 2013; Majid et al., 2014). Additionally, in neurons of patients with AD, Aβ accumulates in autophagic vesicles, further suggesting the involvement of autophagy in AD (Nixon et al., 2005). There is a complex interaction between Aβ and autophagy. In the early stage of AD, the autophagy level of neuronal cells is gradually enhanced, which helps to remove damaged organelles, misfolded proteins and harmful factors, such as IL-1β, L-6, TNF-α, etc. Nevertheless, with the accumulation of abnormal substances, the autophagy-lysosome pathway is gradually blocked, concomitant with the enhanced LC3-II, and impaired binding of autophagosomes to lysosomes, which in turn promotes the development of AD (Switon et al., 2017).

Parkinson’s disease (PD) is the second most common age-related neurodegenerative disease clinically featured by the progressive death of dopaminergic neurons in the substantia nigra. Another pathological hallmark of PD is the deposition of alpha-synuclein-positive protein aggregates termed Lewy bodies, indicating a deficiency in the protein elimination mechanism. Indeed, autophagy failure is recognized as an important pathophysiological mechanism contributing to the initiation and progression of PD. Autopsy of brain tissue from PD patients showed a build-up of autophagosomes, accompanied by the absence of lysosomal markers in dopaminergic neurons, suggesting the impaired autophagy flux (Dehay et al., 2010). Belarbi et al. found that therapeutic activation of autophagy by co-treatment with the mTOR-dependent rapamycin and mTOR-independent trehalose in a PD mouse model successfully alleviates PD symptoms, further implicating the involvement of autophagy in PD (Pupyshev et al., 2019). In addition, both sporadic PD and familial PD can be caused by autophagy-related gene mutations (Abeliovich and Gitler, 2016). For example, loss-of-function mutations in PINK-1 and Parkin, which are essential for mitophagy, cause early-onset autosomal recessive PD (Dorn and Kitsis, 2015). Pathogenic leucine-rich repeat kinase 2 (LRRK2) mutations are the leading cause of familial PD. Saha et al. (2015) found that LRRK2 G2019S mutation in PD patients led to lysosomal aggregation and inhibition of autophagic activity in dopaminergic neurons. The expression of transcription factor EB (TFEB), an important inducer of lysosomal biogenesis and autophagy (Settembre et al., 2011), is remarkably decreased in nuclei of dopaminergic neurons, and co-localizes with Lewy bodies in samples from PD patients (Decressac et al., 2013). In summary, age-dependent neurodegenerative diseases are closely associated with the activity of autophagy.

## Autophagy in the Liver and its Role in Aging

The liver is a vital metabolic organ and possesses high levels of metabolic-stress-induced autophagy. Hepatic autophagy supplies energy to starved cells by breaking down glucose, amino acids and free fatty acids, promoting the synthesis of new macromolecules, and controlling the quality and quantity of organelles like mitochondria. Therefore, autophagy is important for maintaining the balance of energy and nutrient in the liver. A study on perfused rat liver demonstrated that starvation elevated the autophagic proteins degradation rate from about 1.5% of total liver protein per hour (basal) to 4.5% of total liver protein per hour (Schworer et al., 1981). Liver-specific Atg7 deficiency impaired the autophagic protein degradation induced by starvation in the liver and led to hepatic lobules disorders and cell swelling; the mutant mice finally developed serve hepatomegaly (Komatsu et al., 2005). Singh et al. (2009) reported that pharmacological and genetic inhibition of hepatic lipophagy via 3-methyladenine (3-MA) or Atg5 knockdown led to the build-up of lipids drops and triglycerides in mouse liver, emphasizing the requirement of autophagy in liver lipid metabolism. However, hepatocyte-specific deletion of Atg5-deficient promoted hepatocyte proliferation and protected the liver from acetaminophen (APAP)-induced liver injury in mice by sustained activation of Nrf2 (Ni et al., 2012).

Recent studies have shown that the autophagy capacity of older livers is significantly reduced compared to younger livers. Mohammad et al. revealed that the expression of LC3 and the number of autophagic structures declined with age in mice (Uddin et al., 2012). The reduction of autophagy in the aged liver led to decreased ATP synthesis, accumulated lipids and increased oxidative stress, which further compromised the function of the hepatocytes (Stahl et al., 2018). Ogrodnik et al. (2017) demonstrated that hepatocyte aging caused impaired fatty acid oxidation capacity and mitochondrial dysfunction, which in turn promoted lipid accumulation and contributed to hepatic steatosis. Mitochondria are key organelles for lipid metabolism in hepatocytes. The age-related impairment in mitophagy caused reduced mitochondrial turnover and increased mitochondrial dysfunction in the liver, which not only affected lipid metabolism but also induced high levels of ROS production. The excessive ROS further exacerbated the damage to lipid metabolism, ultimately leading to hepatocyte apoptosis and hepatic inflammation (Cao et al., 2016; Chen et al., 2020). Thus, appropriate enhancement of autophagy is protective for the liver. Rapamycin and carbamazepine are commonly known autophagy inducers. Lin et al. (2013) reported that rapamycin and carbamazepine mitigated liver steatosis, liver injury, and dyslipidemia in both alcohol-fed and high fat diet-fed mice by activating autophagy. In contrast, autophagy inhibitors (chloroquine) treatment aggravated hepatic steatosis and liver injury (Lin et al., 2013; Figure 3D).

Unlike other visceral organs, the liver has an amazing ability to regenerate. Liver regeneration is an energy-consuming process that requires an extensive supply of energy for hepatocytes division and growth. Autophagy, especially mitophagy, selectively removes the damaged mitochondria and promotes mitochondrial biogenesis, which decreases ROS production and increases ATP synthesis, providing the environment and energy required for liver regeneration. The regeneration ability of the liver is impaired with age and improving autophagy seems to be a promising way to promote regeneration in aged livers. The mTOR signaling pathway is the most well-known autophagy regulatory pathway. However, mTOR not only regulates autophagy but also plays important role in cell proliferation. Although inhibition of mTOR activity can induce autophagy, it impairs cell proliferation, which is critical for liver regeneration. Therefore, activation of autophagy through an mTOR-independent pathway is a better strategy (Xu et al., 2020). Amiodarone is a common antiarrhythmic medication, which induces autophagy as well. Lin et al. (2015) showed that amiodarone-induced autophagy promoted liver growth and hepatocyte proliferation in an mTOR-independent manner. After partial hepatectomy (PHx), they observed an increased LC3-II and a decreased p62 in amiodarone-treated mice, which were accompanied by elevation of LBWR, Ki-67, PCNA, cell cycle protein levels and reduction of TGF-β1 and p21. Conversely, inhibition of autophagy by chloroquine or Atg7 knockdown exacerbated liver regeneration (Lin et al., 2015).

## Concluding Remarks and Future Perspectives

In summary, autophagy has convincingly been shown to be a prerequisite for longevity and healthspan, and altered autophagy contributes to aging and the transition of the healthy state of several tissues into a presenescent state (Cuervo et al., 2005; Nakamura and Yoshimori, 2018). Although elevation of autophagy has been suggested to have beneficial effects on lifespan and some age-related diseases (Nakamura and Yoshimori, 2018), controversial observations exist regarding the protective and detrimental role of autophagy induction in the context of tissue aging (Schafer et al., 2017). Besides, although autophagy declining with age has been observed in the whole organism and the specific tissues, it remains unclear whether aging causes a decrease in autophagy or vice versa. Thus, there are many challenges in the correlation between autophagy and aging. An in-depth understanding of the underlying mechanisms of autophagy dysfunction in aging in different organs and tissues will shed light on the development of autophagy-targeted therapeutic strategies to combat aging and aging-related disease.

## Author Contributions

HY, YM, and CY prepared the figure. WL, PL, and HY wrote and approved the final text. All authors were involved in the manuscript writing, including discussion of content and writing, and editing of the manuscript.

## Conflict of Interest

The authors declare that the research was conducted in the absence of any commercial or financial relationships that could be construed as a potential conflict of interest.

## Publisher’s Note

All claims expressed in this article are solely those of the authors and do not necessarily represent those of their affiliated organizations, or those of the publisher, the editors and the reviewers. Any product that may be evaluated in this article, or claim that may be made by its manufacturer, is not guaranteed or endorsed by the publisher.
